# ACE2, COVID-19 Infection, Inflammation, and Coagulopathy: Missing Pieces in the Puzzle

**DOI:** 10.3389/fphys.2020.574753

**Published:** 2020-10-06

**Authors:** Zaid Abassi, Abd Al Roof Higazi, Safa Kinaneh, Zaher Armaly, Karl Skorecki, Samuel N. Heyman

**Affiliations:** ^1^Department of Physiology and Biophysics, Rappaport Faculty of Medicine, Technion-Israel Institute of Technology, Haifa, Israel; ^2^Laboratory Medicine, Rambam Medical Center, Haifa, Israel; ^3^Department of Clinical Biochemistry Hadassah Medical Center, Hadassah Hebrew University Hospital, Mt. Scopus, Jerusalem, Israel; ^4^Department of Nephrology, Nazareth Hospital, EMMS, Nazareth and Azrieli Faculty of Medicine in Safed, Safed, Israel; ^5^The Bar-Ilan University Azrieli Faculty of Medicine in Safed, Safed, Israel; ^6^Department of Medicine, Hadassah Hebrew University Hospital, Mt. Scopus, Jerusalem, Israel

**Keywords:** COVID-19 pandemic, angiotensin converting enzyme 2, SARS-CoV-2, RAS inhibition, inflammation, coagulopathy

## Abstract

Engulfed by the grave consequences of the coronavirus disease 2019 (COVID-19) pandemic, a better understanding of the unique pattern of viral invasion and virulence is of utmost importance. Angiotensin (Ang)-converting enzyme (ACE) 2 is a key component in COVID-19 infection. Expressed on cell membranes in target pulmonary and intestinal host cells, ACE2 serves as an anchor for initial viral homing, binding to COVID-19 spike-protein domains to enable viral entry into cells and subsequent replication. Viral attachment is facilitated by a multiplicity of membranal and circulating proteases that further uncover attachment loci. Inherent or acquired enhancement of membrane ACE2 expression, likely leads to a higher degree of infection and may explain the predisposition to severe disease among males, diabetics, or patients with respiratory or cardiac diseases. Additionally, once attached, viral intracellular translocation and replication leads to depletion of membranal ACE2 through degradation and shedding. ACE2 generates Ang 1-7, which serves a critical role in counterbalancing the vasoconstrictive, pro-inflammatory, and pro-coagulant effects of ACE-induced Ang II. Therefore, Ang 1-7 may decline in tissues infected by COVID-19, leading to unopposed deleterious outcomes of Ang II. This likely leads to microcirculatory derangement with endothelial damage, profound inflammation, and coagulopathy that characterize the more severe clinical manifestations of COVID-19 infection. Our understanding of COVID-ACE2 associations is incomplete, and some conceptual formulations are currently speculative, leading to controversies over issues such as the usage of ACE inhibitors or Ang-receptor blockers (ARBs). This highlights the importance of focusing on ACE2 physiology in the evaluation and management of COVID-19 disease.

## Background

Coronavirus disease 2019 (COVID-19), caused by the highly contagious coronavirus 2 (SARS-CoV-2), is initiated by invasion into host cells through viral attachment to angiotensin (Ang)-converting enzyme (ACE) 2. ACE2, expressed in numerous different tissues, serves as an anchor for specific domains on the viral spikes (Hamming et al., 2004; Hoffmann et al., 2020; Zou et al., 2020). Additionally, ACE2, through the modulation of the renin-Ang-aldosterone system (RAS), plays an important physiologic role in the homeostasis of tissue microcirculation and inflammation (Crackower et al., 2002; Hamming et al., 2007; Santos et al., 2008; Clarke and Turner, 2012; Datta et al., 2020). This minireview will address the role of ACE2 within the RAS, and the inter-association of ACE2 and SARS-CoV-2, with their plausible combined impact on the clinical manifestations of COVID-19 disease. We shall further address knowledge gaps that require elucidation in order to better understand the pathophysiology and clinical features of COVID-19 in order to develop effective means for disease prevention and management.

## ACE2: An Important Component of RAS

**Figure 1:**
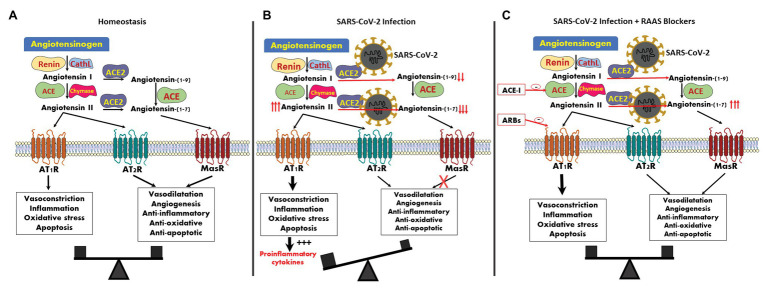
Angiotensin derivatives, their targets and downstream action: **(A)** Balanced impact of angiotensin (Ang) II and Ang 1-7 on vascular tone and control of inflammation. **(B)** SARS-CoV2 infection generates Ang 1-7 depletion, likely leading to unopposed vasoconstriction and inflammation. **(C)** Concomitant renin-Ang-aldosterone system (RAAS) inhibition with Ang-converting enzyme (ACE) inhibitors or Ang-receptor blockers (ARBs) may restore the balance, with parallel suppression of signals mediated by Ang T_1_ receptors (AT1R) and Mas receptor (MasR).

Figure 1A illustrates our current understanding of the complexity of the RAS. Until recently, most clinicians were familiar with only one axis, namely renin-mediated proteolysis and conversion of angiotensinogen to the 10-amino-acid peptide Ang I, followed by a further cleavage by ACE, principally present in the lungs to form the bioactive 8-amino-acid compound Ang II (Crackower et al., 2002; Hamming et al., 2007; Santos et al., 2008; Clarke and Turner, 2012). The COVID-19 pandemic shifted our attention to another component of RAS, namely ACE2, which plays a role in SARS-CoV-2 virulence. Ang II could be further cleaved by ACE2 to form the bioactive 7-amino-acid peptide Ang 1-7. In addition, ACE2 converts Ang I into Ang (1-9), which can be further converted to Ang 1-7 by ACE. A third pathway of Ang 1-7 generation involves neprilysin (neural endopeptidase-NEP), which converts Ang I directly into Ang 1-7 (Tipnis et al., 2000; Crackower et al., 2002; Vickers et al., 2002; Hamming et al., 2007; Santos et al., 2008; Clarke and Turner, 2012). An alternative degradation pathway with conversion of Ang I to Ang II takes place by the proteolytic enzyme, chymase, explaining ongoing generation of Ang II in patients on ACE inhibitors (Miyazaki and Takai, 2006).

Importantly, Ang derivatives differ by their downstream physiologic properties and are mediated by diverse signal transduction mechanisms (Figure 1A). Ang II acts principally as a potent vasoconstrictor, pro-inflammatory, pro-fibrotic, and anti-diuretic agent. These actions are mediated by Ang II binding to Ang T_1_ receptors (AT1R) on affected cell membranes. Opposing activities may be initiated *via* attachment of Ang II to Ang T_2_ receptors (AT2R; Li et al., 2017). Indeed, Ang II-mediated vasoconstriction or vasodilation at the renal cortex and medulla, respectively, reflects diverse receptor distribution and activity, predominantly AT1R in the cortex and AT2R in the medulla (Duke et al., 2003). As also shown in Figure 1A, unlike Ang II, Ang 1-7 exerts unequivocal vasodilatory, anti-inflammatory, anti-fibrotic, and natriuretic actions by binding to a G-protein-coupled Mas receptor (MasR; Li et al., 2003; Santos et al., 2008).

Thus, a tight physiologic balance exists by the opposing effects of Ang derivatives whenever this system undergoes perturbations, with the aim of preventing extreme vasoactive deviations or uncontrolled inflammation and remodeling, with Ang 1-7 serving to counterbalance the undesired adverse effects of unbridled Ang II action.

## ACE2 and SARS-CoV-2 Association

**Figure 2:**
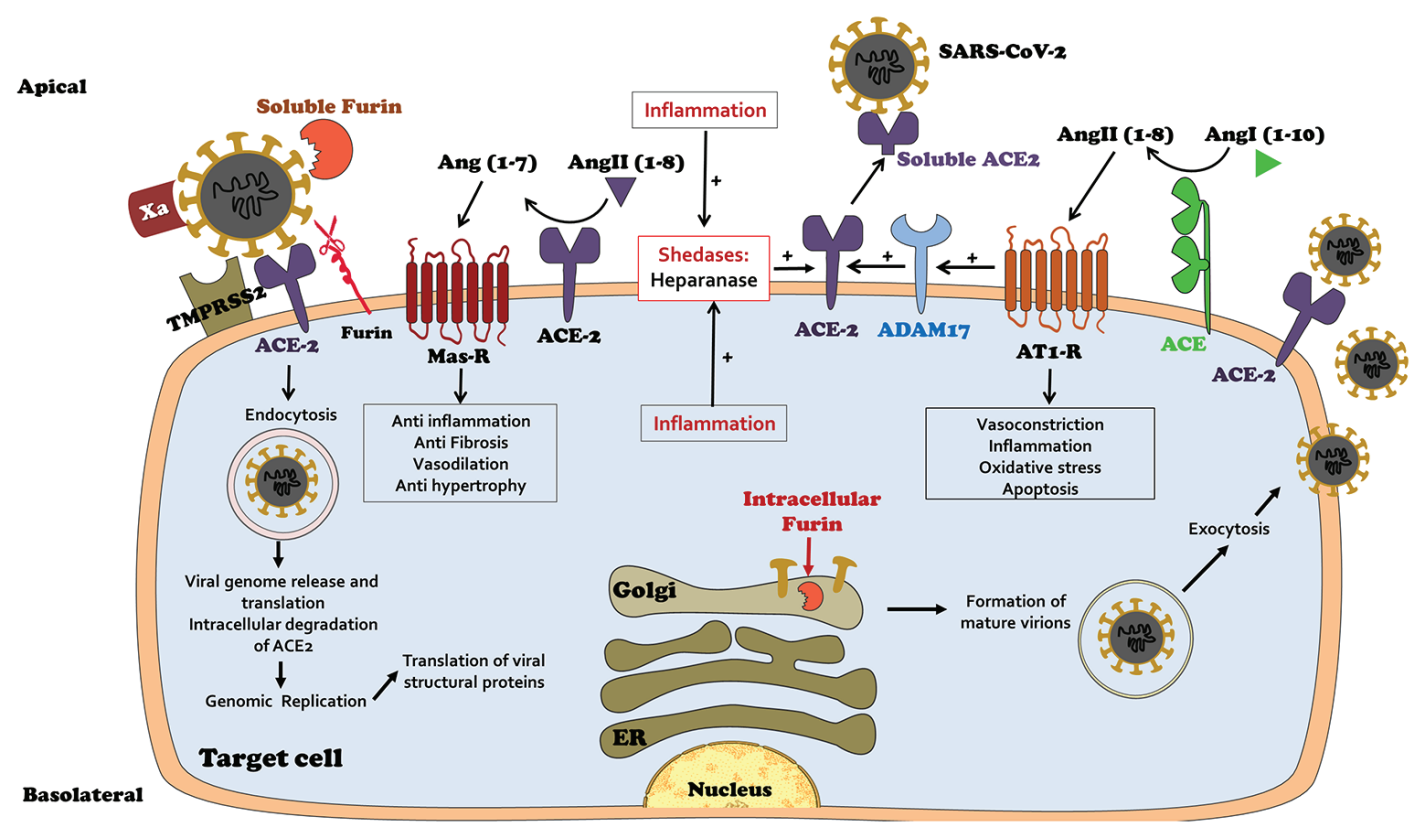
Physiology of coronavirus disease 2019 (COVID 19) homing to target host cells expressing ACE2: viral spike-domains enable attachment to cell-membrane-bound ACE2. Attachment is further enabled by furin, corin, TMPRSS2, and Factor Xa. Following attachment the virus undergoes internalization and replication in host cells, a process associated with degradation of internalized ACE2. Ang 1-7 synthesis consequently declines. Unopposed Ang II action triggers inflammation which activates ADAM 17, leading to shedding of membranal ACE2, further depleting cell-bound ACE2 and local Ang 1-7 synthesis. Viral attachment to target host cells may be attenuated by its competitive binding with rising titers of circulating ACE2.

Angiotensin-converting enzyme is expressed on the plasma membranes of various cell types, including alveolar and intestinal epithelia, vascular endothelial cells in the heart, kidney, and testis, and on macrophages, where it catalyzes the production of Ang 1-7 and its likely paracrine activity (Crackower et al., 2002; Hamming et al., 2007; Santos et al., 2008; Clarke and Turner, 2012; Abassi et al., 2020c). Unfortunately, cell-membrane-bound ACE2 also serves as a binding site for the viral spike proteins of SARS-CoV-1 and SARS-CoV-2 (Li et al., 2003; Hamming et al., 2004; Hoffmann et al., 2020; Walls et al., 2020; Wan et al., 2020; Wu et al., 2020; Zou et al., 2020). The viral attachment to ACE2 with subsequent internalization is facilitated by additional modifications and cleavage of the S1/S2 spike proteins by convertases, such as transmembrane protease serine (TMPRSS 2) and related proteases (Furin and Corin; Heald-Sargent and Gallagher, 2012; Coutard et al., 2020; Hoffmann et al., 2020; Shang et al., 2020; Walls et al., 2020), and probably by activated factor X (Xa), which was shown to cleave recombinant and pseudoviral S protein into S1 and S2 subunits (Du et al., 2007), all exposing the fusion sites in the viral spike protein (Figure 2).

**Figure 3:**
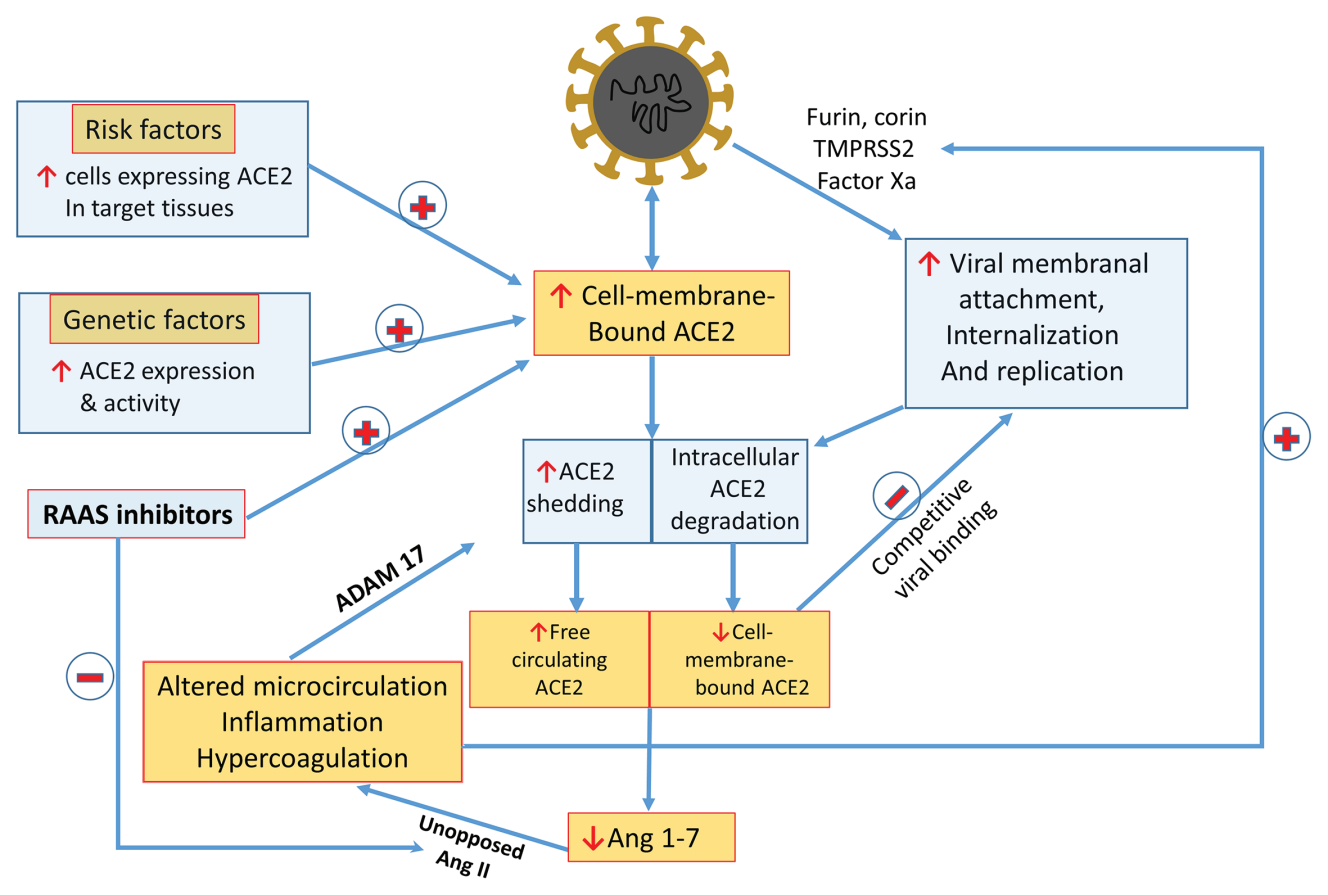
A summarizing scheme of suggested COVID-17/RAS interactions: see text for details. Highlighted are factors enhancing ACE2 expression and viral binding to target host-cells, as are mechanisms leading to declining membranal ACE2 and Ang 1-7 synthesis. The impact of shedded sACE2 on tissue Ang 1-4 production and on inhibiting viral homing to target cells expressing ACE2 by means of competition require further elucidation. RAS inhibitors potentially can enhance viral invasion by enhancing ACE2 expression, yet they may attenuate the unfavorable outcome of Ang 1-7 depletion by a parallel inactivation of functionally opposing Ang II activity. Potential hazardous feed-forward loops are AT1R-mediated enhanced ACE2 shedding and intensification of viral attachment *via* proteases activated by vasoconstriction and ischemia, inflammation, and coagulopathy.

Two principal sites of SARS-CoV-2 invasion include the gastrointestinal and respiratory tracts, which express abundant ACE2. While intestinal homing is clinically more pronounced in children, manifested by gastrointestinal symptoms, the lungs conceivably serve as the principal port of entry, with viral attachment to type II alveolar cells (AT2), and to alveolar macrophages coated by membranal ACE2 (Abassi et al., 2020c,d). Interestingly, conditions identified as predisposing to severe COVID-19 disease are characterized by enhanced pulmonary expression of ACE2. First, chronic airway disease, smoking, and pollution are associated with expansion of the population of alveolar macrophages expressing ACE2 (Abassi et al., 2020d). Furthermore, ACE2 expression is increased in males (La Vignera et al., 2020; Papadopoulos et al., 2020). Indeed, bioinformatics analyses revealed higher abundance of ACE2-expressing AT2 cells in men than women (Wei et al., 2020), potentially enhancing viral susceptibility among men. In this context, testosterone has been described to induce ACE2 expression, the receptor entry of the SARS-CoV-2 infection, but also exerts protective effect against lung injury (Kuba et al., 2005). Enhanced ACE2 is also found in diabetes (Muniyappa and Gubbi, 2020) and heart failure (Zisman et al., 2003; Goulter et al., 2004; Chen et al., 2020), and possibly with the administration of RAS inhibitors (Li et al., 2017). Diabetes is also associated with increased expression of furin (Fernandez et al., 2018). Thus, while testosterone levels decline with aging among men (Harman et al., 2001; Feldman et al., 2002), the presence of comorbidities like obesity, diabetes mellitus, and cardiovascular diseases, possibly counterbalance the decline in viral homing capacity related to age-dependent testosterone drop (Camacho et al., 2013; Rastrelli et al., 2015). In addition, testosterone enhances AT1R expression in male, whereas estrogen preferentially upregulates AT2R expression in females (Chanana et al., 2020). Finally, hypogonadal males are characterized by low T cell count which may provide unrestrained environment for severe responses to SARS-CoV-2 infection (Papadopoulos et al., 2020). In sum, it is tempting to assume that enhanced expression of ACE2 in target organs and also of other molecules permissive to viral binding to ACE2 facilitate viral invasion and augment viral load (Figure 3), although the details of this formulation require validation in further studies.

## Unbalanced RAS in SARS-CoV 19 Disease

As illustrated in Figures 1, 2, SARS-CoV-2 invasion unbalances the RAS. Viral cellular internalization is coupled with degradation of membranal ACE2. Furthermore, circulating Ang II, combined with internalized ACE2 activates a sheddase named ADAM metallppeptidase domain 17 (ADAM 17) also called tumor necrosis factor-α-converting enzyme (TACE; Lambert et al., 2005), which in turn triggers shedding of membranal ACE2 into the circulation with the formation of soluble ACE2 (sACE2), further depleting membranal ACE2 along enhanced TNF-α production (Figure 2). Thus, viral cellular invasion and replication, initially facilitated by ACE2 and in particular under conditions characterized by enhanced ACE2 expression, later lead to diminution of cell membrane-attached ACE2, and likely increase circulating sACE2 (Figures 2, 3). At the microcirculatory and tissue level, this is expected to result in unbalanced paracrine action of Ang compounds, with a local depletion of Ang 1-7 leaving Ang II activity unopposed (Figure 1B). Likely, this has a role in microcirculatory dysfunction, intense inflammation, hypercoagulability, tissue damage, and fibrosis (Figure 3). Lung inflammation in SARS CoV-19 disease exemplifies the outcome of Ang II/Ang 1-7 imbalance: Ang II enhances vascular permeability along infiltration of neutrophils into alveolae and indirectly *via* induction of interleukin 8 (IL-8; Diamond, 2020). Accumulation of neutrophils and their accompanied prooxidative role lead to loss of alveolar epithelial cells and the development of ARDS. Nevertheless, this Ang II-derived lung injury is prevented by Ang 1-7 as was evident in ACE2 deficient mice (Zou et al., 2014).

Additional adverse aspect of unrestricted Ang II action during SARS-CoV-2 infection is the increased tendency of thrombosis documented in large number of hospitalized COVID-19 patients (Bikdeli et al., 2020; Klok et al., 2020). Although this phenomenon is multifactorial, as outlined below, AT1R activation plays an important role where it leads to enhancement of tissue factor (TF) expression on endothelial cells and sequentially initiation of clotting cascade along increased permeability and neutrophils mobilization (Dielis et al., 2005).

## Knowledge Gaps: The Missing Pieces in the Puzzle

Many sections in the preceding paragraphs are based on *in vitro* and animal studies, some with inconsistent and even conflicting interpretations. Furthermore, some fundamental concepts are currently being re-evaluated. For instance, previously reported ACE2 expression on vascular endothelial cells (Hamming et al., 2004) has recently been questioned, based on the measurement of single-cell RNA (Batlle et al., 2020). Human data based on patients infected by SARS-CoV-2 are sparse and are now being intensively studied as we write these lines. It is evident that the foregoing statements should be further examined in the human clinical scenario of COVID-19 disease.

Second, the role of altered ACE2 physiology detailed above in subsequent clinical features of the disease requires in-depth evaluation (Essig et al., 2020). There are several hypothetical mechanisms, outlined in Figure 3, that warrant consideration. Possibly, unopposed Ang II due to depletion of cell membrane-bound ACE2 results in altered regional microcirculation and hypoxia, with the generation of reactive oxygen species and endothelial damage, glycocalyx degradation, and disseminated coagulopathy (Abassi et al., 2020a). This may further compromise the regional microcirculation with a feed forward loop, leading to organ failure including the heart (Abassi et al., 2020b), lungs (Abassi et al., 2020d), and kidneys (Batlle et al., 2020). Furthermore, intense inflammation and coagulopathy may result from unopposed Ang II and by ADAM 17–mediated activation of TNF-α/IL-6/STAT-3 pathways (Hirano and Murakami, 2020) as well as uncontrolled heparanase activity (Li and Vlodavsky, 2009) together with the induction of defensins (Abu-Fanne et al., 2019). Concerning the latter, preliminary findings from our group indicate that alpha-defensin-1, released from polymorphonuclear cells as a part of the inflammatory response, plays a pivotal role in the hypercoagulopathy associated with COVID-19 disease, as its rising titers parallel increasing plasma levels of D-dimers (Higazi AAR, submitted manuscript). Regarding the interplay between Ang II and ADAM 17/TNF-α/IL-6/STAT-3 pathways, it was found that Ang II activates NF-κB and release of proinflammatory cytokines (Dandona et al., 2007; Benigni et al., 2010). Specifically, induction of ADAM17 by Ang II initiates the conversion of interleukin-6 (IL-6Rα) to the soluble form (sIL-6Rα) along activation of signal transducer and activator of transcription 3 (STAT3) *via* the sIL-6Rα-IL-6 complex in various nonimmune cells including fibroblasts, endothelial cells, and epithelial cells (Hirano and Murakami, 2020). Moreover, STAT3, essential for the NF-κB pathway, is principally stimulated by IL-6 during inflammation (Murakami et al., 2019). Since IL-6 plays a key role in the recruitment of lymphoid cells and myeloid cells, including activated T cells and macrophages (Murakami et al., 2019), and likely enhances defensin release (Higazi AAR, unpublished data), its elevated levels during senescence may contribute to the enhanced COVID-19 mortality in aged people and to coagulopathy. Interestingly, AT1R density is increased, while AT2R abundance declines under inflammatory conditions (Diamond, 2020). Collectively, these results may explain proinflammatory cytokine release and hypercoagulopathy during SARS-CoV-2 infection *via* the associated Ang II pathway and a possible therapeutic target *via* the IL-6-STAT3 axis (Diamond, 2020).

Reduced inherent expression of ACE2 in the lungs with aging, as demonstrated in rats (Xie et al., 2006; Alghatrif et al., 2020) may reduce the risk for SARS-CoV-2 infection on the one hand, whereas its further suppression to very low levels during viral infection, on the other hand, could amplify Ang II/Ang 1-7 imbalance, leading to more profound deleterious pulmonary consequences. Conversely, younger individuals with higher inherent ACE2 expression may have a higher incidence, yet less severe SARS-CoV-2 infection, since ACE2 depletion would not be as severe as in aged patients, with Ang 1-7 generation sufficient to counteract Ang II (Alghatrif et al., 2020). Deranged vascular reactivity will likely be affected by other mediators, such as iNOS‐ activation and intense nitric oxide production (plausibly with abundant formation of the toxic-free radical peroxynitrite), and by altered endothelial production of endothelin and prostaglandins. Notably, there are additional plausible inherent feed-forward loops in the scheme of SARS-CoV-2 infection and inflammation, including hypoxia-driven perpetuation of endothelial damage and tissue damage. Furthermore, as illustrated in Figures 2, 3, Ang II suppresses Ang 1-7 generation secondary to downregulation of membranal ACE2 *via* ADAM 17 activation. Moreover, Factor Xa, generated during disseminated coagulation, is expected to expose attachment sites on viral spikes and enhance viral attachment to target cells expressing ACE2 (Du et al., 2007). Interestingly, *in vitro* studies illustrate that heparin interferes with ACE2 binding to the S1 viral spike protein, reducing viral internalization (Mycroft-West et al., 2020). Thus, enhanced heparanase activity in infected patients might damage endothelial cover by heparin-like proteoglycans and further facilitate viral endothelial invasion.

Third, discussions regarding the potential impact of medications affecting RAS are currently based on inconsistent observations and educated guesses (Essig et al., 2020). We really do not know for sure if blocking steps in the RAS cascade indeed results in enhanced ACE2 expression in humans, and whether this promotes viral attachment and invasion. On the other hand, discontinuation of RAS inhibitors might further intensify the uncontrolled action of Ang II, shown in Figure 1B, leaving it unopposed once Ang 1-7 generation is hampered. Those in favor of uninterrupted administration of RAS inhibitors would argue that, as illustrated in Figure 1C, depleting Ang II or blocking its action on AT1R [by ACE inhibitors or Ang-receptor blockers (ARBs), respectively] would balance the exhaustion of Ang 1-7 caused by viral invasion and might prevent consequent vasoconstriction (South et al., 2020). Furthermore, it is also likely that the profile of Ang derivatives may differ in patients treated by ARBs, by ACE inhibitors or by spironolactone (Malha et al., 2020). That is why blanket reassurance regarding continuation of RAS inhibitors during the current pandemic (Vaduganathan et al., 2020) should be regarded with caution. A cautious approach might consider the avoidance of ACE inhibitors or ARBs during an active epidemic in non-infected and hemodynamically-stable patients in order to reduce ACE2 expression, permissive to viral attachment, but consideration of ACE inhibitors, or ARBs at advanced stages of COVID-19 disease to prevent Ang II predominance due to depleted Ang 1-7. Most of the clinical trials and data analysis are performed on adults, however potential differences between adults and children may exist, thus coronavirus-related research should be undertaken in children as well, including the impact of ACE-I and ARBs on COVID-19 evolvement among this subpopulation. Hopefully, this may provide clues for the question why children are at decreased risk of severe COVID-19 disease (South et al., 2020). Furthermore, we have no idea about the function or malfunction of circulating sACE2 following its shedding from cell membranes. Does it exert systemic vasodilation or improve the microcirculation? Can it compete with cell-membrane-bound ACE2 (Ciaglia et al., 2020) and reduce viral attachment to target cells as suggested in Figure 3? Nor can we tell if diverse inherent expression and activity of circulating or cell-bound ACE2 or its capacity to attach to viral spike proteins affects infection, infectivity, or susceptibility to severe and complicated disease. We also are not sufficiently knowledgeable of plausible changes in ACE2 transcription in various tissues in response to SARS-CoV-2 infection. Indeed, Rice et al. (2006) reported that up to 67% of the phenotypic variation in circulating ACE2 could be accounted for by genetic factors. These findings may partially explain the different mortality rate among the various ethnic groups, and strongly support studies of genetic analysis of ACE2 polymorphisms as a reliable approach for precision medicine in the prevention, diagnosis, and therapy of COVID-19 disease. Evidence is currently lacking as to whether levels of circulatory sACE2 may have diagnostic and prognostic implication when monitoring patients infected by SARS-CoV-2, as it does in patients with heart failure (Epelman et al., 2008; Ortiz-Perez et al., 2013). With so many pieces of data missing, the need for vigorous clinical studies guided by physiology-based questions and hypotheses are most urgent. Such a question includes the continuation or even introduction, rather than cessation of RAS inhibitors in patients infected by SARS-CoV-2 (Kai and Kai, 2020), or can we inhibit binding of SARS spike proteins to ACE2, for instance by antibodies, without hampering its catalytic capacities to generate Ang 1-7? Is there a role for the application of Ang 1-7 or MasR agonists or for the administration of intravenous sACE2, with an available proof of concept for such postulated approaches (Yang et al., 2014; Hemnes et al., 2018)?

It is likely that many of the above options will be considered and examined in the near future. Meanwhile, we are challenged by epidemiologic aspects, by issues of supportive and critical care for very sick individuals, and by minimizing the risk to healthcare providers. The ultimate solution probably will be effective vaccination. Yet, until we reach this goal, studying and manipulating ACE2-viral association is a plausible approach, along with the development of effective anti-viral agents.

## Data Availability Statement

The original contributions presented in the study are included in the article/supplementary material, further inquiries can be directed to the corresponding author.

## Author Contributions

ZAb, AH, ZAr, KS, and SH equally participated in the design, execution of the view of point, drafted the manuscript, participated in critical discussions, and revised the manuscript. SK, SH, and ZAb prepared the figures. SH supervised the project. All authors contributed to the article and approved the submitted version.

### Conflict of Interest

The authors declare that the research was conducted in the absence of any commercial or financial relationships that could be construed as a potential conflict of interest.
